# Evidence that coronavirus superspreading is fat-tailed

**DOI:** 10.1073/pnas.2018490117

**Published:** 2020-11-02

**Authors:** Felix Wong, James J. Collins

**Affiliations:** ^a^Institute for Medical Engineering and Science, Massachusetts Institute of Technology, Cambridge, MA 02139;; ^b^Department of Biological Engineering, Massachusetts Institute of Technology, Cambridge, MA 02139;; ^c^Infectious Disease and Microbiome Program, Broad Institute of MIT and Harvard, Cambridge, MA 02142;; ^d^Wyss Institute for Biologically Inspired Engineering, Harvard University, Boston, MA 02115

**Keywords:** COVID-19, SARS-CoV-2, superspreading, extreme value theory, infectious disease

## Abstract

Superspreaders, infected individuals who result in an outsized number of secondary cases, are believed to underlie a significant fraction of total SARS-CoV-2 transmission. Here, we combine empirical observations of SARS-CoV and SARS-CoV-2 transmission and extreme value statistics to show that the distribution of secondary cases is consistent with being fat-tailed, implying that large superspreading events are extremal, yet probable, occurrences. We integrate these results with interaction-based network models of disease transmission and show that superspreading, when it is fat-tailed, leads to pronounced transmission by increasing dispersion. Our findings indicate that large superspreading events should be the targets of interventions that minimize tail exposure.

Superspreading has been recognized as an important phenomenon arising from heterogeneity in individual disease transmission patterns (1). The role of superspreading as a significant source of disease transmission has been appreciated in outbreaks of measles, influenza, rubella, smallpox, Ebola, monkeypox, SARS, and SARS-CoV-2 (1, 2). A basic definition of an *n*th-percentile superspreading event (SSE) has been proposed to be any infected individual who infects more people than does the *n*th-percentile of other infected individuals (1). Hence, if the number of secondary cases is randomly distributed, then for large *n*, SSEs can be viewed as right-tail events. A natural language for understanding the tail events of random distributions is extreme value theory, which has been applied to contexts as diverse as insurance (3) and contagious diseases (4). Here, we apply extreme value theory to empirical data on superspreading in order to gain insight into this critical phenomenon impacting the current COVID-19 pandemic.

## Results and Discussion

We view the number of secondary cases resulting directly from an index case of a disease to be a random variable, *Z.* We also view the individual reproductive number, *v*, to be a random variable representing the expected number of secondary cases caused by an infected individual. Seminal work (1) has suggested that, for SARS-CoV, *Z* follows a negative binomial distribution, *Z∼*negative binomial(*R*_0_,*k*), where *R*_0_ is the basic reproduction number, *k* is the dispersion parameter quantifying variation in transmission, and the mean and variance of *Z* are *R*_0_ and *R*_0_(1 + *R*_0_/*k*), respectively. Assuming that stochastic effects in transmission are modeled by a Poisson process, *v* is gamma-distributed and 1/*k* effectively measures the “flatness” of the distribution of *v*. Different assumptions of the branching process can be modeled, and we focus on the foregoing assumptions for simplicity (1). For SARS-CoV, *k* has been estimated to be ∼0.16 (1); for SARS-CoV-2, *k* has been estimated to be ∼0.1 to 0.6 (2, 5). Importantly, if *Z∼*negative binomial(*R*_0_,*k*), then for *k* ≤ 1, *Z* has an exponential tail (6). This means that the occurrence of SSEs has a probability that decreases exponentially as *Z* increases.

Tails are exceptionally significant in extreme value theory, where they determine how rare extreme events are, how the central limit theorem is generalized, and what distribution the scaled maxima of samples follow. We were therefore interested to determine whether the empirically observed distribution of *Z* for SARS-CoV and SARS-CoV-2 exhibited an exponential tail. We searched the scientific literature for global accounts of SSEs, in which single cases resulted in numbers of secondary cases greater than *R*_0_, estimated to be ∼3 to 6 for both coronaviruses (1, 7). To broadly sample the right tail, we focused on SSEs resulting in >6 secondary cases, and as data on SSEs are sparse, perhaps due in part to a lack of data sharing, we pooled data for SARS-CoV and SARS-CoV-2. Moreover, to avoid higher-order transmission obfuscating the cases generated directly by the index case, we ruled out SSEs where a single infected individual led to a cluster of subsequent infections, but the subsequent infections were not indicated to be secondary cases.

**Fig. 1.**
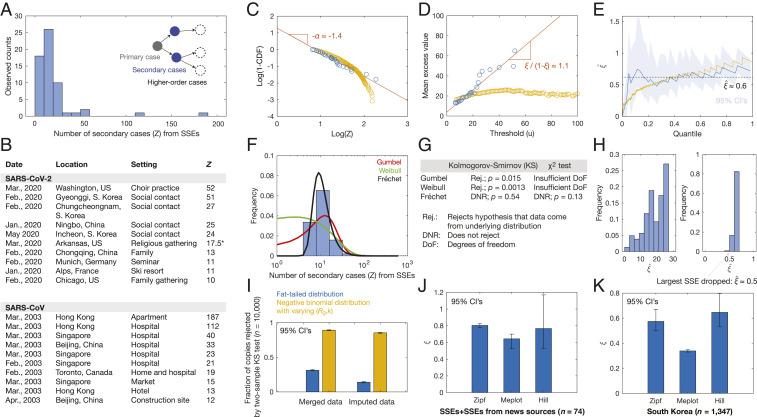
SARS-CoV and SARS-CoV-2 SSEs correspond to fat tails. (*A*) Histogram of *Z* for 60 SSEs. (*B*) Subsample of 20 diverse SARS-CoV and SARS-CoV-2 SSEs. *See Dataset S1 for details. (*C*) Zipf plots of SSEs (blue) and 10,000 samples of a negative binomial distribution with parameters (*R*_0_,*k*) = (3,0.1), conditioned on *Z* > 6 (yellow). (*D*) Meplots corresponding to *C*. (*E*) Plots of $\hat{\xi }$, the Hill estimator for *ξ*, for the samples in *C*. (*F*) Different extreme value distribution fits to the distribution of SSEs. (*G*) One-sample Kolmogorov–Smirnov and χ^2^ goodness-of-fit test results for the fits in *F*. (*H*) Robustness of results, accounting for noise (*Left*) and incomplete data (*Right*). (*I*) Inconsistency of the maxima of 10,000 samples of a negative binomial distribution (yellow) with the SSEs in *A*, accounting for variability in (*R*_0_,*k*) and data merging and imputation, in contrast to the maxima of 30 samples from a fat-tailed (Fréchet) distribution (blue) with tail parameter α = 1.7 and mean *R*_0_ = 3. The numbers of samples in each case were determined so that the sample mean of maxima is equal to the sample mean from *A*. (*J*–*K*) Generality of inferred *ξ* to 14 additional SSEs from news sources (*J*) and a dataset of 1,347 secondary cases arising from 5,165 primary cases in South Korea (*K*) (Dataset S2).

Curating a total of 60 SSEs in this way, we found 45 SSEs associated with SARS-CoV-2 and 15 SSEs associated with SARS-CoV (Fig. 1 *A* and *B*). An additional 14 SSEs were documented in news sources and not scientific studies, and their inclusion does not significantly change the following results, which also hold when accounting for sources of bias (below). Details of the dataset are summarized in Dataset S1.

Several striking observations emerge from the data. While the SSEs surveyed indicated secondary case numbers ranging from ∼6 in a family-spreading incident in Singapore to 187 in an apartment in Hong Kong, many SSEs exhibited significantly more secondary cases than *R*_0_ ≈ 3 to 6, with the conditional sample mean being 19.7 cases (Fig. 1 *A* and *B*).

We next examined the tail behavior of *Z* using inference tools from extreme value theory. We found that the tail of *Z*, as sampled by our list of SSEs, {*Z*_*i*_}, was inconsistent with exponential decay. Instead, we found that the tail of *Z* is consistent with fat-tail behavior using three complementary methods: 1) a Zipf plot; 2) a meplot; and 3) statistical estimators of the tail index, which collectively suggest a power-law scaling of the form Pr(*Z* > *t*)∼*t*^−*α*^ for large *t*, with *α* between 1 and 2 (Fig. 1 *C*–*E* and *SI Appendix*, *Methods*). Equivalently, this observation indicates that the tails of *Z—*as quantified by the threshold exceedance values {*Z*_*i*_ *– u|Z*_*i*_ ≥ *u*}—can be described by the generalized Pareto distribution, with corresponding tail index *ξ* = 1/*α* between 0.5 and 1. That *ξ* ≤ 1 is significant, since all moments higher than 1/*ξ* diverge for a generalized Pareto distribution (3).

Our finding that the tail of *Z* is fat has implications not only for superspreading, but also for modeling the effects of individual variation on disease transmission. First, the fat tail of *Z* makes the distribution of *Z* inconsistent with a negative binomial distribution, and the consistency of the tail with a generalized Pareto distribution suggests that it arises from branching processes in which the time to infection, instead of *v*, is gamma-distributed (so that the tails of *Z* correspond to an exponential-gamma mixture); this prediction is consistent with studies that have fitted serial intervals to gamma distributions (8, 9). Second, since the second moment of *Z* diverges if *α* < 2, the occurrence of SSEs suggests that measuring variances of empirical samples of *Z* can be misleading. Third, fat-tailed distributions generate extreme risk, and superspreading should be mitigated by measures that reduce tail events instead of focusing on the bulk of the distribution.

A complementary way in which we may interpret superspreading is by assuming that SSEs arise not only as right-tail samples of *Z*, but also as the maxima of many samples of the entire distribution of *Z*. The consistency of this viewpoint with the definition of SSEs as right-tail samples of *Z* is given by an important theorem in extreme value theory relating threshold exceedances to extreme value distributions (3). Indeed, SSEs often represent the maxima of values of *Z* observed in transmission clusters. In this case, the Fisher–Tippett–Gnedenko theorem asserts that distributions of the maximum of large numbers of samples converge to either the Gumbel, Fréchet, or Weibull distributions if the tails of the underlying distribution are exponentially decaying, fat, or thin (faster-than-exponential) and finite, respectively. Supporting the view of SSEs as maxima of ensembles of spreading events, we found that the distribution of observed SSEs was consistent with the Fréchet distribution but inconsistent with the Gumbel and Weibull distributions, as measured by maximum-likelihood fitting and one-sample Kolmogorov–Smirnov and χ^2^ goodness-of-fit tests at the 5% significance level (Fig. 1 *F* and *G* and *SI Appendix*, *Methods*).

We next verified that our results were robust to noisy and incomplete data (4). To account for noise, we generated 10,000 copies of the data, where each copy involved multiplying the original data by uniform random variables in [0.5,1.5]—a range that we anticipate to accommodate errors in testing and reporting—and recomputed $\hat{\xi }$ according to the Hill estimator (*SI Appendix*, *Methods*). To account for incomplete data, a random number of observations between 1 and 10 was randomly removed, according to uniform distributions, for 10,000 copies of the data, and $\hat{\xi }$ was recomputed. The variation in $\hat{\xi }$ is summarized in Fig. 1*H*. Notably, we observed that $\hat{\xi }$ was always greater than 0.5, so that the second and higher moments of *Z* diverge.

In a complementary analysis, we tested for sources of bias in the data, which could arise from variations in testing and reporting. As null models, we tested whether the data could be consistent with the maxima of samples from a negative binomial distribution with (*R*_0_,*k*) randomly sampled in [0,6] × [0,1] and in which up to 40% of entries were merged or imputed by the mean. Statistical tests of 10,000 copies of simulated data indicated that these sources of variation cannot explain the observed SSEs, which instead favor an underlying fat-tailed distribution despite this variation (Fig. 1*I*). Moreover, we repeated our analyses after adding 14 SSEs from news sources and for a contact-tracing dataset of 1,347 secondary cases arising from 5,165 cases in South Korea (10) (Dataset S2). We found that both datasets exhibited fat-tailed behavior, with inferred tail indices (*ξ* ≈ 0.3 to 0.8) quantitatively similar to those found above (Fig. 1 *J* and *K*).

**Fig. 2.**
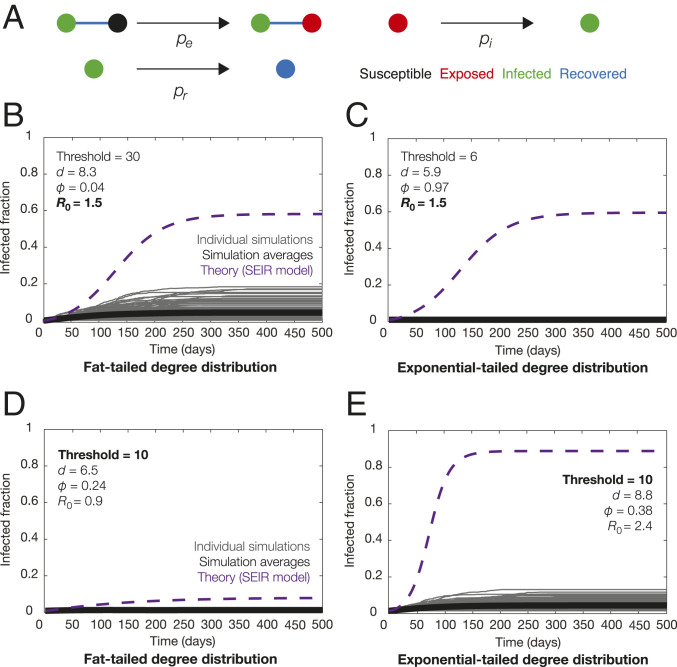
Forward modeling of intervention strategies. (*A*) State transitions in a fine-grained network model of disease transmission. (*B*–*E*) Predicted total infected fraction for an intervention strategy that isolates a fraction *φ* of all individuals, namely those with degree greater than the threshold number, and yielding decreased mean connectivity of *d* and effective basic reproduction number of *R*_0_. Here, *R*_0_ depends on the coefficient of variation of the degree distribution, as detailed in Dataset S3. Trajectories from 100 simulations for BA random graphs (*B* and *D*) and WS random graphs (*C* and *E*) and their averages are shown, compared to the theoretical predictions for a well-mixed model.

Combining these results with modeling can be timely for informing interventions in the current pandemic. As a proof of concept, we considered a network model of transmission which fine-grains an SEIR model (Fig. 2*A*). Here, 1,000 individuals (nodes) each transition between being susceptible (S), exposed (E), infected (I), and recovered or dead (R) with rates $S\overset{\beta SE}{\to }E$, $E\overset{\delta E}{\to }I$, and $I\overset{\gamma I}{\to }R$, as detailed further in Dataset S3, and rates were chosen with *R*_0_ = 3 and a characteristic incubation time of 5 days for SARS-CoV-2 (7). We considered two different graph models with identical mean connectivity (*m* = 10): Barabási–Albert (BA) and Watts–Strogatz (WS), which possess fat-tailed (α = 2) and exponential-tailed degree distributions, respectively. As a simple intervention strategy, we considered node removals in which a fraction *φ* of all nodes is removed starting from those with largest degree. We found that, when the degree threshold for node removals was chosen to yield the same effective value of *R*_0_ in both models, the BA model resulted in greater transmission (Fig. 2 *B* and *C*), indicating that a fat-tailed degree distribution contributes to transmission by increasing dispersion. In contrast, for the same degree threshold, we found that isolating all possible superspreaders—defined here as individuals with degree greater than 10, corresponding to the 80th percentile in the BA model and the 50th percentile in the WS model—suffices to decrease *R*_0_ below 1 and control the pandemic for the BA, but not WS, model (Fig. 2 *D* and *E*). Intriguingly, in both models, stochastic extinction events lead to smaller infected fractions than those predicted by a well-mixed model (Fig. 2 *B*–*E*). These results indicate that transmission is especially pronounced when superspreading is fat-tailed and hint at more detailed models of interventions focused on tail events. We anticipate future models to consider not only heterogeneity in network interactions, but also in infectivity and susceptibility (11).

In summary, we have provided evidence that the distribution of secondary cases, *Z*, is fat-tailed with tail exponent *α* ∈ [1,2]. The fat-tailed nature of *Z* indicates that SSEs have an outsized contribution to overall transmission and should be the targets of interventions that minimize tail exposure, for instance, by preventing large gatherings of susceptible individuals or immunizing select individuals (12). Extreme value theory offers a framework for modeling superspreaders, and we anticipate that using the tools of this theory can, as illustrated here, better allow us to understand the effects of superspreading on the ongoing pandemic.

## Supplementary Material

Supplementary File

Supplementary File

Supplementary File

Supplementary File

## Data Availability

All analysis code are available at GitHub, https://github.com/felixjwong/superspreaders. All study data are included in the article and *SI Appendix*.
