# Supplementary material for: Evidence that coronavirus superspreading is fat-tailed
Source: Proc Natl Acad Sci U S A. 2020 Nov 2;117(47):29416–8. doi: 10.1073/pnas.2018490117 (PMC7703634; doi:10.1073/pnas.2018490117)
Supplement: Supplementary File [file pnas.2018490117.sapp.pdf]

PNAS
www.pnas.org

Supplementary Information for

Evidence that coronavirus superspreading is fat-tailed

Felix Wong[1,2] and James J. Collins[1,2,3,*]

[1]Institute for Medical Engineering & Science and Department of Biological Engineering, Massachusetts Institute of Technology, Cambridge, MA 02139, USA.
[2]Infectious Disease and Microbiome Program, Broad Institute of MIT and Harvard, Cambridge, MA 02142, USA.
[3]Wyss Institute for Biologically Inspired Engineering, Harvard University, Boston, MA 02115, USA.

***Email:** jimjc@mit.edu

This PDF file includes:

Extended Methods
SI References

www.pnas.org/cgi/doi/10.1073/pnas.2018490117

Extended Methods

The Zipf plot shown in Fig.1C of the main text is a log-log plot of the survival function against the number of secondary cases, and the linearly decreasing behavior it shows suggests a power-law scaling of the form $\Pr(Z>t)\sim t^{-\alpha}$ for large t. The value of the power-law coefficient, $\alpha\approx1.45$ (95% CI: [1.38,1.51]), is greater than 1. Equivalently, this observation indicates that the tails of Z—as quantified by the threshold exceedance values $\{Z_i - u | Z_i \geq u\}$—can be described by the generalized Pareto distribution, with corresponding tail index $\xi=1/\alpha\approx0.7$ (95% CI: [0.62,0.76]). That $\xi\leq1$ is significant, since all moments higher than $1/\xi$ diverge for a generalized Pareto distribution (1).

The Zipf plot can be complemented by computing the mean excess function of Z, $e(u)=E(Z-u|Z\geq u)$, which for a generalized Pareto distribution is linear in u with slope $\xi/(1-\xi)$ (1). Hence, checking for linearity in a plot of u against $e(u)$ — a <u>m</u>ean <u>e</u>xcess plot — above some threshold u allows one to verify the existence of fat tails. We observed in a meplot that for $u>10$, $e(u)$ indeed increases approximately linearly with a slope of ~1.11 (Fig.1D; 95% CI: [1.02,1.20]; adjusted R^2: 0.91), suggesting a value of $\xi\approx0.5$, which is qualitatively consistent with the Zipf plot of Fig.1C of the main text.

The Hill estimator of the tail index ξ is

$$\hat{\xi}(k) = \frac{1}{k}\sum_{i=1}^{k} \log\left(Z_{i,n}/Z_{k,n}\right),$$

where $2\leq k\leq n$ and $Z_{n,n}\leq Z_{n-1,n}\leq\ldots\leq Z_{1,n}$ are order statistics of the sample $\{Z_i\}$. Plotting $\hat{\xi}$ against k, we find that the value of $\hat{\xi}\approx0.6$ (95% CI: [0.4,1.0]) observed for a broad range of k is similar to the estimates above (Fig.1E of the main text). We found similar values of $\hat{\xi}$ for two other estimators, the Pickands and Dekkers-Einmahl-de Haan estimators (1,2).

Finally, we note here that a negative binomial distribution of Z, with its exponential tail, would have predicted the distribution of SSEs to be Gumbel-like if each SSE were indeed a maximum of samples of Z. This assertion can be proven by verifying the conditions

$$\lim_{n\to\infty} \frac{\sum_{n}^{\infty} P_j}{\sum_{n+1}^{\infty} P_j} = \text{const.}, \qquad \lim_{n\to\infty} \sum_{n+2}^{\infty} \frac{P_j}{P_{n+1}} - \sum_{n+1}^{\infty} \frac{P_j}{P_n} = 0,$$

where $P_j=\Pr(Z=j)$, sufficient for any discrete distribution to lie in a Gumbel-like domain of attraction (3). Thus, these considerations provide additional evidence suggesting that Z is not negative binomial.

SI References

1. Embrechts, P., Klüppelberg, C., and Mikosch, K. *Modelling Extremal Events for Insurance and Finance.* Springer Stochastic Modelling and Applied Probability (1997).
2. Wong, F. *et al.,* Supporting code for the paper available online at https://github.com/felixjwong/superspreaders.
3. Anderson, C. W. Local limit theorems for the maxima of discrete random variables. *Math. Proc. Camb. Phil. Soc.* **88**, 161-165 (1980).
